# Dietary Intake and Pneumococcal Vaccine Response Among Children (5–7 Years) in Msambweni Division, Kwale County, Kenya

**DOI:** 10.3389/fnut.2022.830294

**Published:** 2022-05-23

**Authors:** Eleonora Migliore, Vivian K. Amaitsa, Francis M. Mutuku, Indu J. Malhotra, Dunstan Mukoko, Anika Sharma, Prathik Kalva, Amrik S. Kang, Charles H. King, A. Desiree LaBeaud

**Affiliations:** ^1^Division of Infectious Disease, Department of Pediatrics, Stanford University School of Medicine, Stanford, CA, United States; ^2^Department of Community Health and Epidemiology, Kenyatta University, Nairobi, Kenya; ^3^Department of Environment and Health Science, Technical University of Mombasa, Mombasa, Kenya; ^4^Vector Borne Disease Control Unit, Ministry of Health, Nairobi, Kenya; ^5^Center for Global Health and Diseases, Case Western Reserve University School of Medicine, Cleveland, OH, United States

**Keywords:** pneumococcal vaccine, dietary intake, vaccine response, malnutrition, child cohort, Kenya

## Abstract

**Background:**

Vaccine and sufficient food availability are key factors for reducing pneumonia outbreaks in sub-Saharan Africa.

**Methods:**

In this study, the 10-valent pneumococcal conjugate vaccine (Synflorix® or PCV10) was administered to a child cohort (5–7 years old, *n* = 237) in Msambweni, Kenya, to determine relationships between dietary intake, nutritional/socioeconomic status of mothers/caregivers, and vaccine response. 7-day food frequency questionnaire (FFQ), dietary diversity score (DDS) and single 24-h dietary recall were used to address participants' dietary assessment and nutritional status. Individual food varieties were recorded and divided into 9 food groups as recommended by Food and Agriculture Organization. Anthropometric measurements, nasopharyngeal swabs and vaccine administration were performed at the initial visit. Participants were followed 4–8 weeks with a blood draw for pneumococcal IgG titers assessed by Luminex assay.

**Findings:**

Chronic malnutrition was prevalent in the cohort (15% stunting, 16% underweight). Unbalanced dietary intake was observed, with mean energy intake 14% below Recommended Dietary Allowances (1,822 Kcal) for 5–7 years age range. 72% of the daily energy was derived from carbohydrates, 18% from fats and only 10% from proteins. Poor anthropometric status (stunting/underweight) was associated with low socioeconomic/educational status and younger mother/caregiver age (*p* < 0.002). Limited intake of essential micronutrients (vitamins A, E, K) and minerals (calcium, potassium) associated with low consumption of fresh fruits, vegetables, and animal source foods (dairy, meat) was observed and correlated with poor vaccine response (*p* < 0.001). In contrast, children who consumed higher amounts of dietary fiber, vitamin B1, zinc, iron, and magnesium had adequate vaccine response (*p* < 0.05). Correlation between higher dietary diversity score (DDS), higher Vitamin E, K, Zinc intake and adequate vaccine response was also observed (*p* < 0.03).

**Interpretation:**

Overall, this study highlights ongoing food scarcity and malnutrition in Kenya and demonstrates the links between adequate socioeconomic conditions, adequate nutrient intake, and vaccine efficacy.

## Introduction

Vaccines are the most cost-effective high-impact public health interventions available, saving millions of lives annually; however, a substantial proportion of childhood deaths (an estimated 2 million each year) are from vaccine preventable illnesses (1). Pneumonia, combined with diarrhea, causes more than 2 million child deaths each year globally (2) and accounts for a very large number of hospital admissions for children of all ages (3).

Sub-Saharan Africa and Southeast Asia bear the greatest burden. Deaths from pneumonia are further aggravated by malnutrition, poverty, and inadequate access to health care (4). Although from 2000 to 2016 the proportion of deaths in young children aged 1–4 years declined in most African regions, sub-Saharan Africa remains the region with the highest under-5 mortality in the world (5). A study conducted between 2007–2010 and associated with influenza surveillance among children with pneumonia have shown how pneumonia was the major cause of hospitalization in children living in rural-costal Kenya setting (6). Though *Streptococcus pneumoniae* is the predominant bacterial etiologic agent in all age groups (7), *Mycoplasma pneumoniae* and *Chlamydia pneumoniae* are also associated with pneumonia in school-aged children and adolescents (5–18 years of age), accounting for up to 50% of all pneumonia cases (8, 9).

The cost of reducing pneumonia deaths treating symptomatic patients by conventional medications is relatively low comparing to other preventions programs promoted in several middle-low-income countries (10), yet implementation of effective preventative interventions such as vaccination remains a challenge in many developing countries, especially regarding distribution of the 10-valent pneumococcal conjugate vaccine (PCV-10). PCV-10 is one of the most expensive vaccines that WHO recommends for inclusion in the National Immunization Programs (NIP). Therefore, the cost for a vaccine campaign can be prohibitive for many low-middle-income countries, like Kenya (11). Despite these difficulties, in 2011 Kenya became one of the first African countries to use the 10-valent pneumococcal conjugate vaccine (PCV-10) (12). The limited evidence available on PCV trials has shown excellent effectiveness of the vaccine (13–15), however immune response can vary widely with different vaccine types and products. The pneumococcal polysaccharide protein D-conjugate vaccine PCV-10 or Synflorix™ is a subunit toxoid-conjugated vaccine that contains ten capsular polysaccharide serotypes from the bacterium *Streptococcus pneumoniae*, eight of which are conjugated to a nonlipidated cell-surface lipoprotein (protein D) of non-typeable *Haemophilus influenzae* (NTHi) and two of which are conjugated to either tetanus or diphtheria toxoid (16). These types of vaccines have superior immunogenicity thanks to the simultaneous presence of the 10 polysaccharide proteins from *S. pneumoniae* and the proteins carriers. This composition, in fact, mimics the type 1 immune response against pathogens like pneumonia and allows the stimulation of the T-cell mediated immune memory response in children under 2 years old of age, reducing at the same time the nasopharyngeal carriage (17).

Age is an important factor that influence vaccine responses, especially in infants where cell-mediated immune responses are less strong, and the response to T-independent polysaccharide antigens is poor (18). A further factor influencing vaccine response is the spacing of vaccine doses. Schedules that have longer intervals between vaccine doses usually lead to higher immune response (19). When PCV-10 vaccine was introduced in Kenya in 2011, the vaccination schedule was in three doses for infants (aged <1 year) and a two-dose catch up campaign for children aged 1–4 years between January and March 2011. Since the good immune response using these temporal intervals, these vaccine schedules are still in use, with a frequency of vaccine administration of 6 weeks apart between three doses and 8 weeks apart between two doses, respectively (20). In addition to age and frequency of vaccine administration, vaccination route is another key factor influencing vaccines responses. For polysaccharide-conjugate vaccines, intramuscular injection leads to higher antibody responses than those obtained by subcutaneous administration (21).

With recent outbreaks of vaccine preventable diseases in Africa, it has become imperative to also assess vaccine efficacy in different local settings. Protection induced by vaccinations is mediated through a complex interplay between innate, humoral, and cell mediated immunity. The range of cofactors that can influence human immune response to vaccination is vast, and include both host and environmental factors, such as age, sex, comorbidities, extrinsic factors like previous infections and preexisting immunity, geographic location, and nutritional status (22).

Several studies investigating interaction between some micronutrients and vaccine responses report conflicting results (23). Although fat-soluble vitamins, such as Vitamin A and D, can affect nearly all aspect of innate and adaptive immunity (24), their role in influencing vaccine response is not clear (25). For example, in children with idiopathic nephrotic syndrome, there is no correlation between Vitamin D levels and antibody responses to pneumococcal polysaccharide vaccine (PPPV23), a vaccine like PCV-10 (26). In contrast, infants receiving supplementation with vitamins A and D have stronger tuberculin skin reaction and lower inflammation responses after Bacille Calmette-Guerin (BCG) vaccination (27). Other animal models and human studies have associated vitamin A and zinc deficiency and disrupted iron metabolism with an impaired ability to mount an effective vaccine response (28–30). In addition, malnutrition has been implicated in the lower efficacy of rotavirus vaccine in low-income countries (31). Globally, child malnutrition is still widespread, causing 3.5 million child deaths every year (32), and this likely poses a significant hurdle to the impact of child vaccination programs.

Kenya's malnutrition rates remain high. About 16% of children aged below 5 years are underweight, 35% are stunted, and 7% are wasted (33). Of particular concern is acute malnutrition, characterized by a sudden, rapid decrease in caloric intake resulting in a reduced weight-for-height *z*-score (WHZ) or in wasting (WHZ < −2) (34). While low WHZ is broadly associated with food insecurity and insufficient caloric and nutritional intake, research has shown that other external variables, such as conflicts and climate conditions, may increase the risk of malnutrition by acting through factors (e.g., reduced agricultural production, reduced access to markets and health/humanitarian aid) more directly related to food security (35, 36). All the current recommended measures of malnutrition have limitations. Weight-for-height z-score (WHZ), together with BMI-for-age Z score (BAZ) and height-for-age Z score (HAZ) are imperfect measures of malnutrition and do not provide a definitive diagnosis (37). Reliance solely on categorical malnutrition parameters (underweight, stunting, wasting) for diagnosis of malnutrition may miss a substantial proportion of children who are below their optimal growth without being wasted (WHZ < -2) (38). Despite these limitations, these measures are meant to identify groups of children who are at the highest risk of mortality and morbidity and thus should continue to be included as a diagnostic criterion for malnutrition. This implies that most children in the developing world are likely to be malnourished when receiving vaccines. However, there is a paucity of studies that adequately examine interactions between dietary intake and vaccine responses.

As previously mentioned, 10-valent pneumococcal conjugate vaccine PCV-10 can stimulate the T-cell mediated immune memory response in infant and young children under 2 years old, in contrast with other non-conjugate polysaccharide vaccines which induce only short-live immune response (17). A long-term study conducted in Kenya between 2002 and 2015 demonstrated how introduction of PCV10 was associated with a reduction in admissions to hospital with clinically defined pneumonia among children aged 2–6 years old (20). Considering the high percentage of malnourished children admitted to hospital with pneumonia diagnosis and that Kenya is ranked among the 15 countries with highest mortality from pneumonia (39), it becomes very important to address the role of nutrition for PCV-10 effectiveness and how certain nutrients can influence immune responses to vaccination. A recent review of both animal and human studies indicates that an insufficient protein intake caused by malnutrition (protein-energy malnutrition or PEM) can impair most aspects of innate immunity, as well as adaptative immunity (24). Another interesting study showed that malnutrition is associated with a decrease in the size of the thymus of infants (source of T-cells), compromising the long-term immunity response and increasing the risk of death in young children (40). Therefore, understanding the association between dietary intake and PCV-10 vaccine response is essential for improvement of pneumonia management and control.

This study focused on establishing the association between dietary intake and antibody response to PCV-10 vaccine among children 5–7 years old in Msambweni division, Kenya. It concurrently assessed demographic and socio-economic characteristics of mothers/caregivers and their 5–7-year-old children, the children's dietary intake, their nutritional status, and the relationship between dietary intake, nutritional status, and response to the 10-valent pneumococcal vaccine.

## Methods

### Ethics Statement and Eligibility Criteria

Ethical clearance was obtained by the Institutional Review Boards at the University Hospital Case Medical Center of Cleveland (protocol number 01-13-13), at Children's Hospital & Research Center Oakland (Protocol Number 2013-020), at Stanford University (Protocol Number 31468), and the Ethical Review Committee of the Kenyatta National Hospital/University of Nairobi (Protocol Number P85/03/2013). Children were eligible if they were residents of the study area since birth, were between 5–7 years old, had provided child assent and written parental consent, and had participated in a previous study cohort (2006–2010) (41–44).

### Study Area and Population

The study population comprised 5–7-year-olds and their caregivers/mothers who had previously participated in a mother-child cohort between 2006 and 2010 (41–44). All the children and their caregivers were inhabitants of Msambweni Division, Msambweni sub-County, Kwale County on the southeast coast of Kenya. The participants were resident among 30 villages within Msambweni Division that surround the Msambweni County Referral Hospital (MCRH) with the majority (72%) of the participants coming from the 7 nearest villages, namely: Bomani, Milalani, Sawasawa, Mwaembe, Vingujini, Kisimachande and Mwishowa Shamba. Msambweni Division has a population of 211,814 distributed in a geographic area of 8,270.3 km^2^ (45). Because of the wide distribution of the population living in Msambweni area, malnutrition remains a serious public health in challenge, with wasting and stunting in each village ranging from 10.4 to 30.8% and 17.6 to 43.0%, respectively (46, 47). A national survey conducted in 2014 at the time of the participants' enrollment indicated stunting, and wasting were highest in the coastal region, at 30.8% and 4.5%, compared to the national average of 26 and 4%, respectively (48). Parasitic infections and co-infections are widespread in the study area (49, 50), and poverty levels are estimated at 35.6% (51, 52). The area experiences bimodal rainfall: long rains occur in March to June and short rains in October to December. Total precipitation varies from 900 to 1,500 mm annually with mean temperatures of 23–34 °C.

### Study Sampling and Subjects

This sub-study was part of a larger study on the effect of early maternal treatment for parasitic infections on vaccine responses (“Enhancing Infant Immunity Study”). A total of 450 children who had participated in a previous study on the effects of fetal malaria (PI King NIH R01AI064687) were eligible for re-recruitment for the present Enhancing Infant Immunity Study (PI King Gates OPP1066865). These children were identified and requested to visit MCRH, and in-home visits were conducted to gather dietary diversity and food intake data. During the initial visit, informed parental consent was obtained; parasitological testing, anthropometric measurements, nasopharyngeal swabs, and vaccine administration were performed. A repeat nasopharyngeal swab and a blood draw for vaccine response testing were done during a follow-up visit 4–8 weeks post the initial visit. Children were vaccinated with the 10-valent pneumococcal vaccine (Synflorix®, GSK, Brussels, Belgium) which contains pneumococcal polysaccharide antigens for SP serotypes 1, 4, 5, 6B, 7F, 9V, 14, 18C, 19F, and 23F. The vaccine was chosen for study of responses by 5–7-year-olds because the children in the previous cohort study had been born before 2011, when the pneumococcal vaccine was not yet given as a standard infant vaccine in Kenya, and so they were not already vaccinated. Thus, it represented a neo-antigen for the cohort, which could better assess their immune response potential. For this in-depth questionnaire sub-study of nutritional effects on PCV-10 vaccine response, only 237 children from the initial cohort of 450 who received the PCV-10 provided follow up blood samples for response testing and were able to complete all the questionnaires provided for this study.

### Dietary Assessment

To characterize the average usual dietary intake of the children, in-home visits were made, and a qualitative 7-day food frequency questionnaire (FFQ) and a single 24-h dietary recall questionnaire (24-HRQ) were applied. The FFQ included locally available foods and was developed and administered to the parents of the children in the study. Frequencies of consuming a particular food were entered either as: daily, 4–6 times a week, 2–3 times a week, once a week, occasionally, or never. The individual varieties of foods consumed were then recorded into the 9 food groups as recommended by Food and Agriculture Organization (FAO) (50). The food groups were starchy staples, meat and fish, dark green leafy vegetables, other vitamin A-rich fruits and vegetables, legumes, nuts and seeds, other fruits and vegetables, milk, eggs, and organ meat. With the 24HRQ, the respondent was asked to describe all the dishes including ingredients that the child consumed in and outside the house in the preceding 24 h. Household food utensils were used to assist study participants to quantify food portions. The amount of nutrients in each ingredient taken by each child was established using Nutri-survey software (EBISpro, Germany) which was modified to include Kenyan recipes.

### Nutritional Status

Nutritional status was determined through standardized/ calibrated anthropometric measurements. Weight and height measurements were carried out for all eligible children according to procedures described by Jeliffe (53). Weight was obtained by digital weight-scale (SECA model 803, Hanover, MD) and was rounded to the nearest 0.1 kg. Height was measured with the use of a stadiometer (SECA model 214, Hanover, MD) and measurements were read to the nearest 1.0 cm. Instruments were calibrated daily prior to use. Every measurement was performed twice, and the mean values used for analysis.

### Vaccine Response Testing

Anti-pneumococcal IgG titers were assessed pre- and post-immunization using a Luminex assay to determine vaccine response as previously described (54, 55). Briefly, we measured anti-pneumococcal IgG levels before and after one dose of the decavalent vaccine. A fluorescent bead immunoassay performed on a MagPix® (Bio-Rad, Hercules, California) system was used for specimen-sparing, multiplex serological testing. Simultaneous measurement of IgG antibody levels against the ten vaccine pneumococcal antigens was obtained as previously described (56, 57). Anti-diphtheria CRM197 levels were also measured to provide an internal control. Dilutions of serum specimens were simultaneously tested for antigen-specific anti-pneumococcal IgGs by adding them to a mixture of pneumococcal polysaccharide carboxyl-coupled (PnPS-coupled) microspheres (MagPlex Beads Bio-Rad, Hercules, California) in a 96-well plate. A subject's individual levels of antigen-specific anti-pneumococcal IgGs were determined from a 5-point standard curve of the median fluorescence intensity (MFI) against expected IgG concentration for 007sp (57) and converted to μg/ml. “Pre-immunity” was defined as having a pre-immunization anti pneumococcal serotype specific IgG titer of > 2 μg/ml (55).

### Statistical Analysis

#### Biological, Demographic, Parasitological, Anthropometric, and Socioeconomic Data

Statistical analyses were conducted using both SPSS for Windows, version 19.0 (SPSS, Inc. Chicago, IL) and SAS for Windows, version 9 (SAS Institute Inc., Cary, North Carolina). For estimation of socio-economic standing (SES), housing conditions and household ownership of selected assets were used to construct an asset index based on principal component analysis (PCA) (58–61). Participating households were divided into two groups (low and high socio-economic standing) according to their scores from the principal component analysis. The assets in the PCA were telephone, radio, television, bicycle, and vehicle, while floor and roof material were used for the housing condition. Other indicators of SES included marital status, primary female caregiver, age of primary female caregiver, source of cooking, drinking, and bathing water, presence of toilet, level of education of primary female caregiver, occupation of primary female caregiver, primary male caregiver source of food, number of children, source of lighting power, and estimated monthly expenditure. The nutritional indicators studied, i.e., standardized BMI-for-age Z score (BAZ), height-for-age Z score (HAZ) and weight-for-age Z score (WAZ) were computed and interpreted using World Health Organization's Anthro-Plus software for ages 5–19 years (WHO, Geneva, Switzerland). Indicator assessment was based on reference growth standards from the year 2006 (59, 60). HAZ is considered an indicator of long-term linear growth, whereas BAZ variations better reflect acute changes in nutritional status. According to WHO standards (61), stunting was categorized as an observed HAZ that was 2 or more SDs below average (HAZ score < −2). Children were categorized as clinically wasted with acute malnutrition if their BAZ was more than 2 SDs below average for their age (BAZ score ≤-2). Children were further identified as severely wasted (chronic malnutrition) if their BAZ was ≤ −3. Cut-offs for BMI-for-age were < −2 (thinness), < −3 (severe thinness), > +1 (overweight), > +2 (obesity) and > +3 (severe obesity).

#### Dietary Diversity and Intake Data

Dietary intake data was analyzed for dietary diversity and adequacy. To estimate how diverse the food eaten by the children was, the Dietary Diversity Score (DDS) was calculated from the 7-day food frequency questionnaire (FFQ). Dietary Diversity Score (DDS) is the number of food groups consumed over a given reference period (62). The individual varieties of foods consumed were recorded into the 9 food groups. If at least one food variety in a food group was consumed within the week a DDS score of 1 was assigned. A DDS score of 0 was assigned if none of the food varieties were consumed within the week (63–65). No consideration was given to the amounts consumed. The obtained DDS was classified into two categories: high diversity (≥7) and low diversity (≤6). Daily energy and nutrient intakes were compared with daily nutrient recommendations based on the Recommended Nutrient Intake values provided by FAO/WHO (66–68). Mann-Whitney non-parametric U test was used to assess statistical differences between energy and nutrient intake and respective Recommended Dietary Allowances (RDAs). Amounts of energy and nutrients consumed that were below 80% of the recommended amounts were considered inadequate, while those equal to or above 80% of the RDAs were considered adequate (69). The 20% allowance was used because the Dietary Reference Intakes (DRIs) are designed with a risk allowance that allows for individual physiological fluctuations of nutrient needs to be met. Thus, intakes just below the reference can't be assumed to be inadequate, as a reference value itself is not a strict threshold. The list of macro and micronutrients that were included for assessment of adequacy were chosen based on their public health relevance and the likely availability of nutrient values in food composition tables.

Chi-square testing was used to assess the effect of dietary diversity intake (DDI) and SES on nutritional status (stunting and underweight). A multivariate logistic regression model was also used to explain the association between observed growth levels and demographics data, including mother/caregiver age and education, SES, and nutritional status. Vaccine response was compared using a 2-sided, 2-sample t-test to identify differences in specific macronutrients between low responders (anti-pneumococcal IgG titers ≤ 2ug/ml) and high responders (anti-pneumococcal IgG titers ≥ 2 ug/ml) to the vaccine.

## Results

### Socio-Demographic Characteristics of Children and Their Caregivers

A total of 237 children aged 5–7 years were studied. Compared to girls, more boys participated in the study, but the difference was not significant (55.34 vs. 44.74%). The mean age of the children was 6.0 ± 0.6 years (range 4.8–7.4 years). Mothers were the primary caregivers for 88%, with a mean age of 31.8 ± 5.9 years (range 19.0–50.0 years). The other caregivers group consisted of grandmothers and aunts. Among the study participants' households, the mean number of children per household was 4.3 ± 1.6 (range 1.0–9.0 children).

Supplemental Table 1 shows the socio-demographic characteristics for participating children and their caregivers in Msambweni (see Supplementary Material section). Most of the caregivers were married while the remainder were single, separated, or widowed. Nearly half of the mothers were housewives, about one-third were running small businesses, 9.34% were salaried workers, 8.41% were small-scale farmers and the rest, 1.32%, were casual laborers or a community worker. Most of the mothers had a low education level. Only a few had attained secondary education or higher (8.94%), while a majority (91.1%) had not gone to school or had attained primary education only. Not shown, the asset index based on housing conditions and household ownership of selected assets was used to classify 50.2 % of the study children as coming from households with relatively lower socio-economic status and the rest coming from households with higher socio-economic status.

### Dietary Diversity

The caregivers reported the children in the study had consumed 34 food varieties in the week preceding the food frequency questionnaire (FFQ) administration. Supplemental Table 2 summarizes food groups and food items used by participating children in Msambweni Division during the one week before their household survey (see Supplementary Material section). Overall, all children in the study had consumed starchy staples and meat and fish in the week preceding the survey. The other commonly consumed food groups were dark green leafy vegetables and other vitamin A rich fruits and vegetables. The usual diet of the children consisted mainly of starchy staples and the most consumed starchy staples were maize, rice, and wheat flour-based products. The starchy staples were commonly taken together with fish, beans, and cabbages. The children's diet was poor in flesh products but was rich in fish. Fish was the only food that was frequently consumed in the meat and fish food group. Fish, pawpaw, kales (Sukuma-wiki), mangoes, oranges, and bananas are some of the foods that are locally available and are therefore consumed either moderately or more frequently. On the other hand, chicken, and chicken eggs, though locally available, are rarely eaten. The children's usual diet was exceptionally poor in milk, eggs, and organ meat food groups.

### Dietary Intake

Intakes of energy and selected nutrients are described in Table 1. Although the mean percent daily energy intake from carbohydrates (72%) was above the DRI range of 45–65%, the overall mean energy intake (1,584 kcal) for the study children was below their RDA (1,822 kcal). This was because energy intake from other major sources of energy, i.e., fat and proteins, was suboptimal.

**Table 1:** Mean (SD) daily energy and nutrient intake compared to RDAs among participating 5–7-year-old children (*N* = 237) in Msambweni, Kenya.

**Nutrients**	**Mean (SD)**	**RDA**	**>80%**	**FAO/WHO**
			**RDA (%)**	**NRVs (2012)**
Energy (kcal)	1,583.7 (372.6)	1,822.2 (142.0)	151 (63.7)	1,810 (0)
Carbohydrates (g)	291.8 (61.0)	250.5 (19.5)	222 (93.7)	248 (0)
Protein (g)	40.9 (10.7)	16 (2.5)	236 (99.6)	50 (0)
Fats (g)	32.1 (21.5)	60.7 (5.0)	38 (16.0)	65 (0)
Dietary fiber (g)	25.5 (7.4)	25.0 (0)	179 (75.3)	25 (0)
Vitamin B1 (mg)	1.2 (0.4)	1.0 (0)	210 (89)	0.9 (0)
Vitamin B2 (mg)	0.7 (0.2)	1.0 (0)	89 (38)	1.2 (0)
Niacin (mg)	11.3 (3.5)	8.0 (0)	219 (92.4)	15 (0)
Vitamin B6 (mg)	1.3 (0.4)	1.0 (0)	213 (89.9)	1.3 (0)
Total folic acid (μg)	182.4 (107.8)	200 (0)	107 (45.2)	200 (0)
Vitamin C (mg)	62.5 (56.9)	25.0 (0)	180 (76.0)	100 (0)
Vitamin E (mg)	0.6 (0.5)	7.0 (0)	0 (0)	9 (0)
Vitamin K (μg)	19.7 (23.4)	55 (0)	21 (8.9)	60 (0)
Potassium (g)	1.6 (0.5)	3.8 (0)	4 (1.7)	3.5 (0)
Calcium (mg)	144.6 (67.1)	1,000.0 (0)	0 (0)	1,000 (0)
Iron (mg)	11.3 (3.3)	10.0 (0)	198 (83.5)	14 (0)
Zinc (mg)	6.6 (1.6)	5.0 (0)	226 (95.4)	8.4 (0)
Magnesium (mg)	396.6 (103.5)	130 (0)	237 (100)	310 (0)
Phosphorus (mg)	843.1 (213.2)	500 (0)	233 (98.3)	700 (0)

*Data as means ± SD. P-value shows comparison between the mean nutrient level, RDA value and FAO/WHO recommended nutrient intakes*.

The mean percent energy intake from protein (10%) was low, but within the DRI range of 10–35%, while the mean percent energy from fat (18%) was below the DRI range of 20–35%. Only 63% of the children have consumed in 1 day an adequate amount of food for their energy needs. The mean intake of dietary fiber was not statistically different from the RDA values, meaning that most children (75%) were consuming foods that met their dietary fiber needs. Based on daily mean intake, the study children were observed to have consumed adequate or more than adequate vitamin B1, vitamin B3 (niacin), vitamin B6, vitamin C, zinc, iron, magnesium, and phosphorus. The mean intake of vitamin B2, vitamin K, vitamin E, total folic acid, potassium, and calcium did not meet the RDA (*p* < 0.001). No participant in this study met the RDA for calcium due to extremely low dairy product intake.

### Nutritional Status

Table 2 summarizes the anthropometric findings of the children in the study. Generally, both acute and chronic malnutrition were reported. Chronic malnutrition as reflected by stunting (HAZ < −2) was prevalent with 15% (35/237) of studied children stunted. Underweight (WAZ < −2) was the most pronounced form of acute malnutrition with over 16% (39/237) of the surveyed children having low weight-for-age. Only a small proportion of the children studied were wasted (4%) or overweight (2%) at time of the study. Boys were more malnourished than girls, but differences were not significant for stunting, wasting, or underweight status.

**Table 2:** Anthropometric indicators of Malnutrition for children 5–7 years in Msambweni, Kenya (*n* = 237).

**Indicator**	**Boys**	**Girls**	**All**
	**(*N =* 131)**	**(*N =* 106)**	**(*N =* 237)**
**Height –for- age score (HAZ)**			
Mean	−1.11	−0.97	−1.04
Standard deviation	1.02	0.95	0.99
Stunted (HAZ < −2) §	16.79%	12.26%	14.77%
**Weight- for –age (WAZ)**			
Mean	−1.12	−0.98	−1.06
Standard deviation	0.97	0.91	0.95
Underweight (WAZ < −2) ¥	18.32%	14.15%	16.46%
**BMI- for-age (BAZ)**			
Mean	−0.6	−0.61	−0.6
Standard deviation	0.91	0.8	0.86
Overweight (1 < BAZ <2)∨	2.29%	1.89%	2.11%
Wasted (BAZ < −2)∧	4.58%	3.77%	4.22%

*Data as means ± SD. §STUNTED defined by WHO Criteria: Height-for-age < −2; ¥ UNDERWEIGHT defined by WHO Criteria: Weight-for-age < -2; ∨ OVERWEIGHT defined by WHO Criteria: BMI-for-age +1 < BAZ < 2; ∧ WASTED defined by WHO Criteria: BMI-for-age < −2*.

Table 3 shows the relationship between maternal education, mother or caregiver age, dietary diversity score and socioeconomic status. Of the 117 stunted children, 81(69%), and 36 (31%) were categorized in the low and high dietary diversity categories, respectively.

**Table 3:** Relationship of participant undernutrition with reported maternal education, mother or caregiver age, child's dietary diversity score, and household socioeconomic status.

Predictors*		**Stunting**			**Underweight**		
		**Stunted (*N =* 117)**	**Normal (*N =* 107)**	***P*-value**	**Underweight (*N =* 95)**	**Normal (*N =* 107)**	***P*-value**
Maternal education^*^							
	Primary and below	102	93	0.818	82	93	0.799
	Secondary and above	11	9		9	9	
Mother/caregiver age^*^							
	Young (<30years)	48	38	0.397	37	38	0.614
	Old (≥30years)	69			58	69	
Dietary diversity score^*^							
	Low DDS	81	76	0.769	66	76	0.809
	High DDS	36	31		29	31	
Social-economic status^*^							
	Low SES	69	43	0.005	58	43	0.003
	High SES	48	64		37	64	

* *Predictors (Maternal education data, Mother/caregiver age, Dietary diversity score data and Social-economic status) data are missing in different subjects. Numbers reported in the table don't add up to the total number of identified subjects (N value). P-value shows comparison between predictors and stunting/underweight values. Definitions of Dietary diversity score and Social-economic status are given in the text*.

Chi-square analysis showed that stunting was positively associated with low socioeconomic status; 59% (69/117) of the stunted children belong to the low SES group (*p* = 0.005). Similarly, underweight children (61%, 58/95) were found significantly more often among those with low SES background (*p* = 0.003). Mother/caregivers age and maternal education were not statistically significantly associated with either stunting or underweight.

A multivariate analysis (logistic regression) was performed in Table 4 to further investigate the association between stunting and underweight (as outcome variables) adjusted for maternal education, mother/caregiver age, Dietary Diversity Score, and household socioeconomic status (as predictor variables).

**Table 4:** Multivariate analysis (logistic regression) of the association between a child's stunted or wasted status and maternal education, mother or caregiver age, dietary diversity score, and socioeconomic status.

	**Stunting**			**Underweight**		
Predictors	**Estimate**	**Standard error**	***P*-value**	**Estimate**	**Standard error**	***P*-value**
Mother/caregiver age	−0.226067	0.285178	0.428	−0.0194	0.2902	0.947
Maternal education	0.432489	0.478033	0.367	0.48046	0.48586	0.323
Socioeconomic status (low vs. high)	−0.828457	0.283259	0.003	−0.088232	0.29104	0.002
Dietary diversity score (low vs. high)	−0.003849	0.299344	0.99	−0.10935	0.30539	0.72

*Data as means ± SD. P-value shows comparison between predictors and stunting/underweight values. Definitions of predictors (Maternal education data, Mother/caregiver age, Dietary diversity score data and Social-economic status) are given in the text*.

Data show again that stunting and underweight, considered as outcome variables, are significantly correlated with a low socioeconomic status (predictor variable, *p* = 0.002), confirming the bivariate results using the Chi-square analysis.

### Dietary Intake and Pneumococcal Vaccine Response

The general trend observed was that all children who consumed in 1 day the Recommended Nutrient Intake of different nutrients (Table 1) responded adequately to PCV-10 vaccination. Table 5 shows children who responded adequately to the vaccine (high responders, anti-pneumococcal IgG titers ≥ 2ug/ml) consumed daily higher amounts of food rich of dietary fiber, vitamin B1, zinc, iron, and magnesium (*p* ≤ 0.05), compared to the children who responded inadequately to PCV-10 vaccination (low-responders, anti-pneumococcal IgG titers ≤ 2ug/ml).

**Table 5:** Mann-Whitney U statistical analysis of PCV-10 vaccination response and dietary intake.

**Nutrients**	**Low responders**	**High responders**	***W*-value**	***P*-value**
	**Median, IQR (*N =* 106)**	**Median, IQR (*N =* 64)**		
Energy (kcal)	1,528.955 (396.5325)	1,648.515 (437.785)	4,104.5	0.02202
Carbohydrates (kcal)	284.765 (66.2525)	303.37 (88.185)	4,040.5	0.03715
Protein (g)	39.33 (11.075)	41.58 (13.365)	3,286.5	0.1628
Fat (g)	28.375 (22.48)	30.585 (17.605)	3,942.5	0.0769
Dietary fiber (g)	24.66 (10.4825)	27.495 (9.2425)	4,141	0.01607
Vitamin B1(mg)	1.105 (0.515)	1.26 (0.4925)	4,042	0.03669
Vitamin B2 (mg)	0.705 (0.3175)	0.755 (0.325)	3,980	0.05878
Niacin (mg)	11.035 (4.66)	12.195 (4.65)	3,984	0.05712
Vitamin B6 (mg)	1.175 (0.525)	1.285 (0.5075)	3,822.5	0.1666
Folic acid (g)	142.47 (102.015)	167.105 (92.215)	3,816.5	0.1727
Vitamin C (mg)	54.8 (63.9125)	42.405 (66.8575)	3,317	0.8106
Vitamin E (mg)	0.38 (0.3675)	0.43 (0.585)	3,797.5	0.1926
Vitamin K (g)	12.07 (18.1625)	13.515 (15.96)	3,903.5	0.09843
Potassium (g)	1,494.67 (605.4)	1,656.89 (587.155)	3,902.5	0.1009
Calcium (mg)	125.705 (73.82)	131.12 (78.735)	3,607	0.4903
Iron (mg)	11.095 (4.215)	12.205 (3.7775)	4,164	0.01309
Zinc (mg)	6.445 (2.065)	7.08 (2.0675)	4,256.5	0.005454
Magnesium (mg)	395.89 (140.915)	422.735 (115.76)	4,050	0.03446
Phosphorous (mg)	831.72 (237.9525)	887.815 (273.4175)	3,974.5	0.06123

*Data as median values with their 25%–75% interquartile ranges. P-value shows comparison between nutrients and PCV-10 vaccine response. Definitions of low responders and high responders are given in the text*.

The logistic regression shown in Table 6 also confirmed the correlation between daily intake of certain micronutrients and PCV-10 vaccine response. Data from both (Table 1 and Table 6) confirm that children who had in 1 day limited intake of fat, vitamins E, and K, folic acid, potassium, and calcium responded poorly to the PCV-10 vaccination (*p* < 0.03), highlighting that a higher Dietary Diversity Score and the intake of these nutrients can be correlated with adequate vaccine response.

**Table 6:** Multivariate analysis (logistic regression) between vaccine response and dietary intake.

	**Vaccine Response**		**P Value**
**Predictors**	**Estimate**	**Standard Error**	
Mother/caregiver age (<30 or ≥30)	0.347	0.413	0.401
Maternal education (Primary and below or secondary and above)	−0.740	0.413	0.352
Social-economic status (Low vs. High)	−0.094	0.397	0.819
Dietary diversity score (Low vs. High)	−0.942	0.439	0.032
Carbohydrates (g)	−0.004	0.012	0.740
Protein (g)	−0.093	0.137	0.498
Fat (g)	0.026	0.019	0.187
Dietary Fiber (g)	0.095	0.135	0.482
Vit B1 (mg)	1.277	5.942	0.830
Vit B2 (mg)	−0.157	3.936	0.968
Niacin (mg)	0.073	0.492	0.883
Vit B6 (mg)	−1.666	1.476	0.259
Folic Acid (mg)	0.004	0.008	0.587
Vit C (mg)	−0.003	0.008	0.749
Vit E (mg)	3.407	1.538	0.027
Vit K (μg)	−0.072	0.035	0.032
Potassium (mg)	0.054	0.002	0.976
Calcium (mg)	0.002	0.007	0.763
Iron (mg)	−0.638	0.389	0.101
Zinc (mg)	1.964	0.718	0.006
Magnesium (mg)	0.007	0.017	0.690
Phosphorus (mg)	−0.007	0.013	0.556

*Data as means ± SD. P-value shows comparison between predictors, nutrients, and PCV-10 vaccine response. Definitions of predictors (Maternal education data, Mother/caregiver age, Dietary diversity score data and Social-economic status) are given in the text*.

## Discussion

Although the preliminary results of this study are encouraging and emphasize the association between SES, educational status, nutritional status, and vaccine response, there are limitations that require to be addressed in the future with more detailed projects. Since Msambweni Division and the related villages are widely distributed along a vast geographic region, it was not possible to collect at the time of enrollment/initial visit data related to biochemical markers of nutrient status, or to do multiple 24-h dietary recalls. As already reported from previous studies conducted in Kenya (70, 71), our results indicate that among our participants in Msambweni, there is an association between reduced food intake and reduced vaccine response for specific foods and food groups. Similar patterns have been already seen in other studies (72), which suggest that lower intake of certain micronutrients may be the cause of lower vaccine response.

Summarizing the data obtained from our participants, overall, the children in this cohort had a mean energy intake ~14% below the recommended RDA (1,584 Kcal reported average vs. 1,822 Kcal recommended). Data from both the 7-day food frequency questionnaire (FFQ) and the single 24-h dietary recall method (24-HRQ) showed that the diets of the study children were predominantly based on starchy staples with little or no animal products and few fresh fruits and vegetables. 72% of the daily energy was derived from carbohydrates, while only 18% was derived from fats and 10 % from proteins. On average, there was a limited intake of essential fats and essential minerals like calcium in their diets. As reported, only 16% of the participants got in 1 day at least 80% of the recommended fats RDA for children between 5–7 years old, while no one among the participants met the recommended calcium RDA for that age range.

Diets lacking Animal Source Foods (ASF) are associated with inadequate micronutrient intake (34, 42, 73) and are invariably related to low quality diets. It was thus not surprising that the diet of this study cohort provided inadequate intake of fat, vitamins (A, B2, K, and E) and minerals (folic acid, potassium, and calcium). Poor anthropometric status (stunting and underweight) was generally associated with low socioeconomic status, low educational status of mothers and caregivers, which are all factors indicative of poor household food security as was the case in other studies. Children of mothers who did not complete the primary school or who had no formal education were more likely to be stunted than children of mothers with a secondary or higher education (74). The present study confirmed this condition also in the Msambweni division, with a statistically significant correlation between maternal age, SES, malnourishment, and stunting (*p* < 0.002). Chronic malnutrition was prevalent in the study cohort, as indicated by stunting (15%) and underweight (16%). Prevalence of wasting (4%) was within acceptable levels of >5%. The level of malnutrition reported in this study was comparable with earlier studies in the region (75, 76).

Antibody (IgG) titer data collected from our study cohort have shown a substantial variation between individuals in the immune response to PCV-10 vaccination, as previously observed in other similar studies (77). As already mentioned before, a limited intake of certain nutrients may play an important role in immune responses to vaccination (78), especially for vaccines such as polysaccharide-conjugate vaccine PCV-10 which stimulate cellular immune response (T-cell dependent). Several studies have already highlighted how adequate nutrients intake at the time of the vaccination can influence the level of antibody responses (79, 80).

Our data have a similar pattern which have been already seen in other study populations. Children with a better SES and a better daily nutrient intake have higher level of PCV-10 antibody (IgG) titer after vaccine inoculation, compared with children with malnourishment and stunting. In addition to socio-economic status and maternal factors, this study highlights how individual dietary intake factors can be associated with lower PCV-10 antibody titer, thus lower vaccine response.

Children in Msambweni division have limited intake of essential micronutrients (vitamins A, E, K) and minerals (calcium, potassium) in a day, due to poor consumption of fresh fruits, vegetables, and animal source foods (dairy, meat). This finding can be correlated with low PCV-10 antibody titer (*p* < 0.001). This pattern is well known for fat-soluble vitamins, especially for Vitamins A and E which can affect nearly all aspect of innate and adaptative immunity when their levels are in deficiency (24).

Mineral deficiency is also correlated with lower immune response to vaccination. Studies with humans have shown that deficiency of dietary zinc resulted in thymic atrophy, impairing the production of T-cell and as consequence the cellular immune response (23). Similarly, our data showed a correlation between a daily lower intake of some micronutrients and the PCV-10 vaccine response in terms of PCV-10 antibody titer. This includes fiber intake, Vitamins B1, E, K, Iron and Zinc (*p* < 0.05).

A comparable data trend has been seen in recent study conducted in Kenyan infants, where anemia and iron deficiency at the time of vaccination predicted a decreased response to diphtheria, pertussis, and pneumococcal vaccines, and that a primary response to measles vaccine may be increased by iron supplementation at time of vaccination (78).

Higher Dietary Diversity Score (DDS) is also correlated with the PCV-10 vaccine response outcome in the Msambweni division (*p* < 0.03), confirming the possible impact of a reduced daily food intake in the vaccine efficacy. Despite the trends noted in those data, it is still uncertain whether malnourishment directly affects the vaccine efficacy. Several micronutrients play an important role in the cellular immune response in which polysaccharide-conjugated vaccines like PCV-10 are involved, however results from related studies are often conflicting (81).

Putting together the data from this study and from the current literature, it is evident that a lower nutritional intake can be a critical determinant of immune response (82). In fact, children malnourishment is currently considered as the most common cause of secondary immunodeficiency worldwide (83). Furthermore, despite childhood malnutrition is not often considered or diagnosed as a comorbidity involved in several infectious diseases, it affects both innate and adaptive immunity. The term “nutritionally acquired immunodeficiency syndrome” (NAIDS) has been recently put forward, highlighting how micronutrient sufficiency plays a major role in determining immune response and immune modulation (84).

In conclusion, this study has shown the effect of lower socio-economic status, food insecurity, and lack of fundamental education on children's health in the rural Msambweni division in Kenya. There are limitations in this study, including unavailability of continuous dietary variables to better detect the scalar effects of dietary intakes and growth deficits, as well as better clinical assessment in terms of biochemical markers. These approaches could be used in the future with a larger cohort sample size. Overall, all the results collected from this study emphasize the need to address food insecurity in children living in Kenyan rural communities, as well as the need for a serious investment in major new research to delineate the nature, strengths, and mechanisms of interactions between nutrients and the specific and nonspecific responses to a vaccination. Therefore, this study can be considered an exploratory approach that can generate new hypothesis regarding the links between nutrition and response to conjugate vaccines.

## Data Availability Statement

The raw data supporting the conclusions of this article will be made available by the authors, without undue reservation.

## Ethics Statement

The studies involving human participants were reviewed and approved by Internal Review Board of Stanford University (IRB#31468), Institutional Review Boards of University Hospital Case Medical Center of Cleveland, Children's Hospital (IRB#01-13-13) and Research Center Oakland (IRB#2013-020), and by the Ethical Review Committee of the Kenyatta National Hospital/University of Nairobi (P85/03/2013). Written informed consent to participate in this study was provided by the participants' legal guardian/next of kin.

## Author Contributions

EM and VA participated in conceptualization of the study, analysis, interpretation of the data, and drafting of the manuscript. FM and IM were major contributors to study conceptualization, study design, and provided oversight for study procedures performed in Kenya. DM contributed to study design and coordinated study procedures in Kenya. AS, PK, and AK participated in acquisition and analysis of the data and revising of the manuscript. CK and AL were major contributors to study conceptualization, study design, data interpretation, and provided substantial revision to the manuscript. All authors have read and approved the final submitted manuscript.

## Funding

Supported by the Bill and Melinda Gates Foundation, Seattle, WA (Bill and Melinda Gates Foundation Healthy Growth Program Grant, OPP1066865; Enhancing Infant Immunity: Effect of Early Maternal Treatment for Parasitic Infections; PI: Charles King). The funding sources had no role in the design of the study, collection, analysis, or interpretation of data, nor did they have a role in writing the manuscript.

## Conflict of Interest

The authors declare that the research was conducted in the absence of any commercial or financial relationships that could be construed as a potential conflict of interest.

## Publisher's Note

All claims expressed in this article are solely those of the authors and do not necessarily represent those of their affiliated organizations, or those of the publisher, the editors and the reviewers. Any product that may be evaluated in this article, or claim that may be made by its manufacturer, is not guaranteed or endorsed by the publisher.

## Abbreviations

WHO: World Health Organization
UNICEF: United Nation Children's Fund
KNBS: Kenya National Bureau of Statistic
PEM: Protein-Energy Malnutrition
KEMRI: Kenya Medical Research institute
CDC: Center for Diseases Control and Prevention
MCRH: Msambweni County Referral Hospital
FAO: Food and Agriculture Organization
FFQ: 7-day food frequency questionnaire
24-HRQ: 24 hour recall questionnaire
PCV-10: 10-valent pneumococcal conjugate vaccine
IgG: Immunoglobulin G
SES: Socioeconomic status
PCA: Principal component analysis
HAZ: Height-for-age Z score
WAZ: Weight-for-age Z score
BMI: Body Mass Index
BAZ: BMI-for-age Z score
DDS: Dietary Diversity Score
DRI: Dietary Reference Intake
RDA: Recommended Dietary Allowances
ASF: Animal Source Food
SD: Standard Deviation.
