# Supplementary material for: Dietary Intake and Pneumococcal Vaccine Response Among Children (5–7 Years) in Msambweni Division, Kwale County, Kenya
Source: Front Nutr. 2022 May 23;9:830294. doi: 10.3389/fnut.2022.830294 (PMC9169235; doi:10.3389/fnut.2022.830294)
Supplement: Supplementary file 1 [file Data_Sheet_1.PDF]

Supplementary Material

<u>Supplemental Table 1</u>: Socio-demographic characteristics for participating 5–7-year-old children and their caregivers in Msambweni, Kenya

Socio-demographic characteristics	Mean ± SD	Group Percentage
**Child characteristics (n=237)**		
Age in years (5-7 years)	6.0 ± 0.6	
Male		55.34%
Female		44.74%
**Mother as primary caregiver (n=232)**		
Age in years	33.9 ± 8.8	
Number of children	4.3 ± 1.6	
**Marital status**		
Married		84.88%
Single		6.75%
Separated		6.75%
Widowed		1.76%
**Occupation**		
Business/petty business		31.62%

Salaried worker		9.34%
Farmer		8.41%
Others		1.32%
**Education**		
No schooling		45.66
Primary		45.62
Secondary School or higher		8.94%

Data as means ± SD. Percentages are related to a total of 237 children's participants and 232 related mothers/caregivers living among 30 villages within Msambweni rural area. Each socio-demographic characteristic is represented as percentages.

Supplemental Table 2: Food groups and food items used by participating children in Msambweni Division during the one week before their household survey

Food groups	Frequency of use	Mean number of food items	Percentage of children using the food group
**Starchy staples**	100%	2.6	100% Maize
	75% Rice		
	73% Wheat flour		
	16% Cassava		
	1% Millet		
**Meat and fish**	98%	1.5	95% Fish
	27% Beef		
	28% *Rastrineobolaargentea* (Omena cyprinid fish)		
	5% Goat meat		
	1% sheep meat		
**Dark green leafy vegetables**	86%	2.1	51% Kales
	46% Cabbage		
	16% Cowpea leaves		
	9% Spinach		
	7% Pumpkin leaves		
	1% Amaranth		

**Other vitamin A rich fruits and vegetables**	88%	1.5	74% Mango
	37% Pawpaw		
	38% Orange		
	4% Pumpkin		
**Legumes, nuts, and seeds**	76%	1.3	68% Beans
	26% Ground nuts		
	17% Green grams		
	14% Cowpeas		
	9% Lentils		
**Other fruits and vegetables**	71%	1.1	51% Banana
	37% Lemon		
	4% Pineapple		
	18% Watermelon		
**Milk**	54%	0.6	54% Cow Milk
	2% Goat Milk		
**Eggs**	33%	0.4	32% Chicken eggs
	4% Duck eggs		
**Organ meat**	2%		2% Liver

Data represent food groups and food items (means) used by children in Msambweni division one week before the survey. Percentages indicate frequency of consumption of each food group per number of participants.
